# Extracorporeal shockwave therapy for the treatment of lower limb intermittent claudication: study protocol for a randomised controlled trial (the SHOCKWAVE 1 trial)

**DOI:** 10.1186/s13063-017-1844-4

**Published:** 2017-03-06

**Authors:** Thomas Cayton, Amy E. Harwood, George E. Smith, Joshua P. Totty, Daniel Carradice, Ian C. Chetter

**Affiliations:** Academic Vascular Surgery Unit, Hull and East Yorkshire Hospitals NHS Trust, Hull Royal Infirmary, Anlaby Road, Hull, HU3 2JZ UK

## Abstract

**Background:**

Peripheral arterial disease (PAD) has a population prevalence of 4.6% with intermittent claudication (IC) presenting as one of the earliest and most common symptoms. PAD has detrimental effects on patients’ walking ability in terms of maximum walking distance (MWD) and pain-free walking distance (PFWD). Research has suggested extracorporeal shockwave therapy (ESWT) may induce angiogenesis in treated tissue; therefore, our objective is to assess the tolerability and efficacy of ESWT as a novel treatment of intermittent claudication.

**Methods/design:**

Patients with unilateral claudication will be randomised to receive either ESWT (PiezoWave 2 shockwave system) or sham treatment to the calf muscle bulk three times per week for 3 weeks. All patients are blinded to treatment group, and all assessments will be performed by a masked assessor. Treatment tolerability using a visual analogue scale, ankle-brachial pressure index, MWD, PFWD and safety will all be formally assessed as outcome measures at baseline and at 4, 8 and 12 weeks follow-up.

**Discussion:**

This trial will be the first of its kind in terms of methodology in relation to ESWT for intermittent claudication. A double-masked randomised controlled trial will provide useful information about the potential for the use of ESWT as a non-invasive treatment option and the need for further robust research.

**Trial registration:**

ClinicalTrials.gov, NCT02652078. Registered on 17 October 2014.

**Electronic supplementary material:**

The online version of this article (doi:10.1186/s13063-017-1844-4) contains supplementary material, which is available to authorized users.

## Introduction

Peripheral arterial disease (PAD) is common in western society with a prevalence estimated at 20% of those aged over 60 [1]. Intermittent claudication (IC), a manifestation of PAD in the lower limbs, causes sufferers to experience cramping pain in the affected muscles, most commonly the calves, which occurs after walking a short distance and increases in intensity until the person stops, where the pain then gradually subsides. PAD can be limiting or occasionally debilitating and has been shown to have considerable deleterious effects on patients’ quality of life [2].

There is a 15-fold increase in mortality secondary to cardiovascular disease in symptomatic patients with severe large vessel PAD; therefore, treatment is based around minimising cardiovascular risk factors and reducing risk of disease progression [3]. With conservative management strategies a proportion of patients will remain with stable symptoms, whilst a minority will deteriorate and require intervention in the form of an endovascular or open vascular surgical procedure.

The physiological negative effects caused by PAD are hypothesised to be due in part to recurrent episodes of ischaemia-reperfusion injury (IRI), which occurs as claudicants exercise (ischaemia) and rest (reperfusion). IRI causes progression of atherosclerosis, as well as endothelial dysfunction and end organ damage [4–6]. During ischaemic episodes and subsequent reperfusion, leucocytes become activated, resulting in release of proteolytic enzymes, cytokines and reactive oxygen species [7, 8].

## Background

A shockwave is described as a transient short-term acoustic pulse with a high peak pressure. This waveform generation and rise to peak pressure occur within nanoseconds [9]. Biological effects of shockwaves on human tissue were first documented during World War II when severe lung injuries secondary to water bombs were noted within casualties who did not display any evidence of external injury [10]. The medicinal use of high energy extracorporeal shockwaves did not develop until the 1980s, when they were targeted to fragment urinary stones (lithotripsy); results led to this technique becoming the gold standard treatment option for urolithiasis [9]. From this point the developing use of extracorporeal shockwave therapy (ESWT) followed an interesting course which started after an incidental finding of iliac bone thickening in patients undergoing lithotripsy, which proposed the potential osteoblastic effect of shockwave therapy [11]. Researchers confirmed these observations through a number of clinical trials using shockwave therapy on delayed non-union fractures [12–14]. Observational improvements to soft tissue wounds overlying these fractures treated with ESWT then encouraged research into the benefits of shockwave therapy in hard-to-heal chronic soft tissue wounds [15].

The exact mechanism of action of ESWT on soft tissue is not fully understood, although the principle of mechanotransduction is thought to be responsible for the biological changes that take place. Mechanotransduction is the transfer of mechanical stimuli into chemical signals; this takes place at a cellular level where mechanosensory components within the cell wall are stimulated, which activates gene expression of growth factors and cytokines as well as cell proliferation or differentiation [16]. The mechanical force is a secondary effect of cavitation which can be visualised in water by the creation of cavitation bubbles that then emit secondary shockwaves. This physical force can also be seen when shockwaves are directed over a thin aluminium foil, which show the effect of cavitation bubbles collapsing on or near the foil [17].

Increased expression of growth factors such as endothelial nitric oxide synthase (eNOS) and vascular endothelial growth factors (VEGFs) have been demonstrated following treatment of soft tissues with ESWT during various studies both in vivo and in vitro. However, the majority of this evidence is from animal studies, and further investigation is required [18–21].

Due to these findings, further applications of ESWT are beginning to be explored. One such application included the use of ESWT to treat ischaemic heart disease where focussed shockwaves were directed at ischaemic areas of myocardium. This demonstrated some positive findings with notable results including improvement in left ventricular ejection fraction (LVEF), ventricular wall thickening, reduced symptoms, reduced nitroglycerine use and again increased upregulation of growth factors and angiogenesis [22]. A further use has potentially been found with small trials commencing in ESWT for peripheral vascular disease, which has led to the development of this current protocol.

### Summary of current evidence

To date, four studies have investigated ESWT in claudicants (Table 1). They include one randomised control trial of 22 patients with 12 patients receiving treatment and 10 receiving a sham control. This study targeted ESWT at a stenotic plaque lesion within lower limb arteries and demonstrated significant reduction in degree of arterial stenosis, significant improvement in pain-free walking distance (PFWD) and reduction in pain severity. These findings were also coupled with a significant reduction in Fontaine staging [23].

**Table 1:** Human studies related to ESWT for peripheral arterial disease

Study/author(s)	Type	Sample size	Methods	Significant results	Limitations
Ciccone MM, Notarnicola A [23]	Randomised control trial	*N* = 22, cases = 12, controls =10	4 sessions at 1 weekly intervals, 2000 impulses, 0.03 mJ/mm^2^. Aimed at stenotic lesion	Increased *pain-free walking distance* and reduction in pain severity. Reduction in degree of arterial stenosis. Reduced Fontaine staging	Small sample size, short-term follow-up
Tara S, Miyamoto M [24]	Prospective non-randomised pilot study	*N* = 10	6 sessions (3 times per week for 2 weeks). 6 spots per session (300 impulses/spot) 0.11–0.12 cmJ/mm^2^	No adverse events. Improved transcutaneous oxygen tension (TcPO_2_) in both calf and dorsum of foot. Scintography demonstrated improved perfusion in dorsum of foot only	Small sample size, non-randomised, short-term follow-up
Belcaro G, Cesarone MR [25]	Prospective non- randomised	*N* = 32	6 sessions (3 times per week for 2 weeks) 1000 impulses per session, 0.08–0.43 mJ/mm^2^	Improved *pain-free walking distance*, analogue scale for pain, laser Doppler skin perfusion, partial pressure of oxygen and decreased partial pressure of carbon dioxide. Reduced ORACLE score	Small sample size, non-randomised short-term follow-up
Serizawa F, Ito K [26]	Prospective non-randomised	*N* = 12	9 sessions (3 times per week for 3 weeks) 40 spots per session with 200 impulses/spot 0.1 mJ/mm^2^	Improved *maximum walking distance*, walking impairment questionnaire scores and shorter recovery time	Small sample size, non-randomised, short-term follow-up
Serizawa F, Ito K [26]	Prospective non-randomised	*N* = 6	6 sessions (3 times in first week (days 1, 3 and 5) and 3 sessions in week 5 (days 29, 31, 33) 40 spots per session with 200 impulses/spot 0.1 mJ/mm^2^	The *maximum walking distance* was significantly increased at 4 weeks and 8 weeks follow-up	Small sample size, non-randomised, short-term follow-up

Data captured in italic are significant outcome for measures in the trial protocol

Another study was a small prospective non-randomised pilot study of 10 patients which aimed to prove safety; its primary end point was observation of adverse events. This study found no adverse events secondary to shockwave therapy. The secondary end point was a measure of tissue perfusion using scintography, laser Doppler and measurement of transcutaneous oxygen tension (TcPO_2_). The use of scintography demonstrated significantly improved tissue perfusion in the dorsum of the foot; however, perfusion was unchanged in the lower leg region. Maximum TcPO_2_ increased significantly in both the calf region and dorsum of the foot. Skin perfusion pressure measured using the laser Doppler showed increases in both regions, but neither was significant [24].

Another prospective study of 32 patients underwent EWST for critical limb ischaemia with both rest pain and localised distal necrosis. Outcomes included PFWD, visual analogue scale (VAS) for pain, laser Doppler skin perfusion, partial pressure of oxygen and partial pressure of carbon dioxide. All of these outcomes demonstrated significantly improved measurements [25].

Finally a further two prospective non-randomised trials by Serizawa et al. with a total of 6 patients and then 12 patients with Fontaine stage II PAD were treated with ESWT and demonstrated significantly improved maximum walking distance, shorter recovery time and improved walking impairment questionnaire scores [26].

### Aims and objectives

This study aims to compare the effects of extracorporeal shockwave therapy (ESWT) to the use of a sham control group on walking distances in subjects with lower limb intermittent calf claudication.

#### Primary outcome

The primary outcome is the effect of extracorporeal shockwave therapy in comparison to sham treatment on the maximum walking distance (MWD) — the distance covered before the subject is unable to continue — at baseline and at 4 weeks, 8 weeks and 12 weeks post therapy.

#### Secondary outcomes

The secondary outcomes are as follows:

- Claudication distance, i.e. distance covered prior to onset of any symptoms, at baseline and at 4 weeks, 8 weeks and 12 weeks post therapy
- Ankle-brachial pressure index (ABPI), measured at baseline and at 4 weeks, 8 weeks and 12 weeks post therapy
- Patient reported walking distance at baseline, 4 weeks, 8 weeks and 12 weeks post therapy
- Quality of life as measured by Short Form 36 questionnaire (SF-36) at baseline, 4 weeks, 8 weeks and 12 weeks post therapy
- Quality of life as measured by the EuroQol 5 domains questionnaire (EQ-5D) at baseline, 4 weeks, 8 weeks and 12 weeks post therapy
- Subject tolerance of treatment, measured by VAS after each individual treatment session

### Design

This is a single-centre, double-blinded randomised controlled trial comparing extracorporeal shockwave therapy (ESWT) to sham treatment in the management of intermittent calf claudication.

### Setting

The trial will be conducted in the Academic Vascular Surgical Unit of the Hull York Medical School, based at Hull Royal Infirmary (University Teaching Hospital). All procedures will be performed in an outpatient-based or day-case setting.

### Patient identification

Patients will be recruited from vascular outpatient clinics and assessed for inclusion by the research team against the listed inclusion and exclusion criteria. In order to achieve target sample size, all patients identified as suffering from PAD will be screened for inclusion.

### Target population and recruitment

#### Inclusion criteria

Patients will be included who:

- Have unilateral or bilateral intermittent calf claudication (stable for the last 3 months)
- Are able to give written informed consent to participate in the study
- Are >18 years old
- Are able to adhere to the protocol and attend all follow-up appointments
- Are currently receiving ’best medical therapy’ — antiplatelet and statin medication

#### Exclusion criteria

Patients will be excluded if they:

- Currently have a malignancy
- Are allergic to or intolerant of either antiplatelet medication or statin therapy
- Are pregnant (pregnancy test performed at screening if necessary)
- Have a metal implant near the treatment area
- Are taking anticoagulation medication (i.e. warfarin)
- Are diagnosed with a blood clotting disorder

### Screening and evaluation

Patients identified as potential participants will undergo a screening interview.

Baseline data will be collected from all consenting participants. Data collected will include:

- Evaluation of compliance with inclusion and exclusion criteria
  - Issue of Patient Information Sheet if not already issued
- Informed consent (24 hours later)
- Participant’s name and address and, if used by the participant, mobile telephone number and email address (for the receipt of follow-up questionnaires) and date of birth
- Details on participant’s general practitioner
- Demography and medical history
- Physical examination to include palpation of lower limb pulses
- Ankle-brachial pressure index (ABPI)
- Vital signs
- Assessment of any concurrent medications
- Pregnancy test if necessary
- Body mass index (BMI)
- Smoking history
- Baseline quality of life

Appropriately screened and consented individuals will be further assessed as follows:

1. Baseline visit
  - Patient reported walking distance (PRD)
  - Vital signs
  - Quality of life
  - Treadmill assessment to report intermittent claudication distance (ICD) and maximum walking distance (MWD)
    For treadmill walking distances, ICD and MWD will be measured on a treadmill at 2.5 km/h at a 10° incline for up to 10 minutes. Participants will be asked to rest for 30 minutes prior to testing to ensure a resting state and negate effects of mobilising to the vascular laboratory.
    Randomisation will occur at the end of the baseline visit via sealed envelope software to either active treatment or control group by a second investigator. The treatment will then proceed as follows:
    (e) The second investigator will then perform ESWT therapy for the active group and sham treatment for the control group. Sham treatment will be delivered by setting the generator to off and replicating the sound of the active treatment via an audio recording emitted from the machine
    (f) All patients will then complete a tolerability questionnaire for the ESWT treatment.
2. Weeks 1, 2, 3 visits
  - Vital signs
  - ESWT performed by second investigator with pulsewave generator on for active group and off for control group
  - Patient to complete tolerability questionnaire for ESWT treatment
  - PRD recorded
  - Assessment of any concurrent medications
  - Assessment of any adverse events
3. Week 4, 8, 12 visits
  - PRD
  - Vital signs
  - Quality of life
  - Treadmill assessment to report ICD and MWD
  - Assessment of any concurrent medications
  - Assessment of any adverse events

## Method/design

### Trial treatments/study procedures

Patients on the treatment arm of the trial will receive ESWT using the PiezoWave2 shockwave system, in accordance with the manufacturer’s instructions and unchanged from its current clinical use at 100 impulses 0.1 mJ/mm per cm^2^. Trained staff will provide all treatments, including sham treatment. Both local research and development and medical physics departments deem this device low risk and safe to use. The device will be targeted at the gastrocnemius muscles of the affected lower leg for several minutes at each treatment session. Participants in the control group will undergo an identical process as if treatment were being given but with the shockwaves not being administered and the sham treatment described above alternatively used.

1 shockwave session = 100 impulses per cm^2^ covering two areas per affected leg, with one (6 cm × 5 cm) on the medial head of the gastrocnemius and one (6 cm × 5 cm) on the lateral head of the gastrocnemius. This calculates as 6000 impulses/5 Hz/0.1 mJ/mm^2^ in total.

### Environmental conditions

A designated clinical room in the vascular laboratory at Hull Royal Infirmary will be used for all examination and treatments. The device will be applied to the gastrocnemius muscle of the affected limb/s at two sites (medially and laterally). One course of treatment will consist of three separate treatment sessions per week on alternate days for 3 weeks.

### Outcome measurements

#### Primary outcome

The primary outcome is the short-to-medium term effect of ESWT in comparison to sham treatment on MWD in patients suffering intermittent calf claudication at baseline, 4 weeks, 8 weeks and 12 weeks post therapy.

#### Secondary outcomes

The secondary outcomes are as follows:

- Claudication distance (CD) — the distance covered prior to onset of any symptoms — at baseline, 4 weeks, 8 weeks and 12 weeks post therapy
- Pre and post treatment ABPI at baseline, 4 weeks, 8 weeks and 12 weeks post therapy
- Patient reported walking distance (PRD) at baseline, 4 weeks, 8 weeks and 12 weeks post therapy
- Acceptability and tolerability of the treatment measured by VAS after each individual treatment session
  - Control group patients will be asked to complete tolerability questionnaires as if they were receiving active treatment.
- Quality of life as measured by SF-36 at baseline, 4 weeks, 8 weeks and 12 weeks post therapy
- Quality of life as measured by the EQ-5D at baseline, 4 weeks, 8 weeks and 12 weeks post therapy

### Assessments

**Fig. 1:**
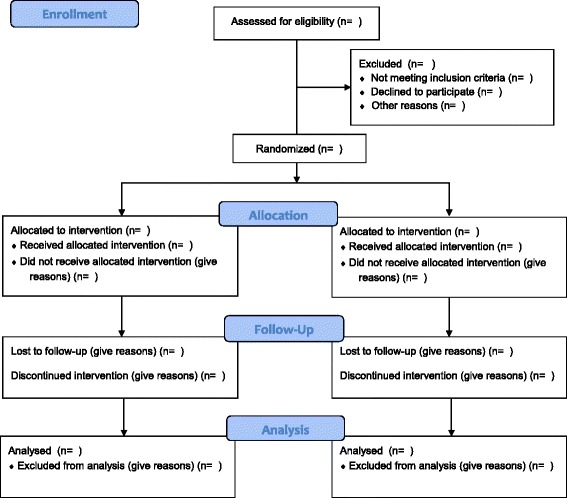
Consolidated Standards of Reporting Trials (CONSORT) flow diagram

Following completion of the consent procedure, baseline data will be collected as defined above. Outcome measurements will then be recorded according to the subsequent study visits. See Fig. 1 and Table 4.

#### Assessments prior to the first ESWT therapeutic session

Assessments prior to the first EWST session are:

- PRD
- ABPI
- Claudication distance
- Maximum walking distance
- Quality of life questionnaires (SF-36 and EQ-5D)

#### Assessments during all therapeutic treatments

During all therapeutic treatments, we will assess:

- Pain rating of treatment using VAS
- Quality of life questionnaires (SF-36 and EQ-5D)

#### Assessments during weeks 4, 8 and 12 follow-up appointments

Assessments at weeks 4, 8 and 12 are:

- Tolerability questionnaire (week 4 only)
- PRD
- ABPI
- Claudication distance
- Maximum walking distance
- Quality of life questionnaires (SF-36 and EQ-5D)

### Concurrent medications

The study will not influence clinical management of the patient; therefore, there are no restrictions of prescribed medication. Researchers will monitor concomitant medications taken by the patients at screening and during the entire study period.

### Adverse events

Serious adverse events will be reported to the Sponsor and the National Research Ethics Service (NRES) recognised Research Ethics Committee (REC).

### The device

The device being used in this trial is the PiezoWave 2. Training and support are provided by Impact Medical, Liverpool, UK.

### Safety and tolerability

The device is a CE-marked (CE 93/42/EEC) British Standards Institute, notified body 0086), commercially available device. Trained staff will manage the device and are responsible for applications of treatment. The facilities where participants will be assessed are fully equipped for all emergency situations.

### Safety reporting

The safety definitions are given below.

*Adverse event* (*AE*): An AE is any untoward medical occurrence in a subject to whom a medicinal product has been administered as part of a research study, including occurrences which are not necessarily caused by or related to an investigational medicinal product (IMP).

*Adverse reaction* (*AR*): An AR is any untoward and unintended response in a subject to an IMP which is related to any dose administered to that subject.

Important medical events that may not be immediately life-threatening or result in death or hospitalisation but may jeopardise the patient or may require intervention to prevent one of the other outcomes listed in the definition above should also be considered serious.

*Serious adverse event* (*SAE*): An SAE is an event not related or unlikely to be related to the IMP. An adverse event becomes serious if it:

- Results in death
- Is life-threatening
- Requires hospitalisation or prolongation of existing hospitalisation
- Results in persistent or significant disability or incapacity
- Is a congenital anomaly or birth defect

*Serious adverse reaction* (*SAR*): A SAR is a serious event which is suspected (possibly, probably or definitely) to be related to an IMP and expected for the IMP.

*Suspected unexpected serious adverse reaction* (*SUSAR*): A SUSAR is a serious event which is suspected (possibly, probably or definitely) to be related to an IMP and unexpected for the IMP, i.e. not previously documented in any of the IMP information (investigator brochure, Summary of Product Characteristics, patient information leaflet) or protocol on device deficiencies.

*Device deficiency*: A device deficiency is an inadequacy of a medical device with respect to its identity, quality, durability, reliability, safety or performance. All device deficiencies will be recorded and reported accordingly.

*Anticipated and unanticipated serious adverse device effects* (*SADE*/*USADE*): There are no known SADEs with this device; therefore, all SADEs are, by definition, unanticipated.

If a USADE is suspected, no additional patients will be enrolled until the cause of the event and its relationship to the study device have been determined. If the event is device-related, the following action will be necessary. If there is a specific type of patient who is vulnerable to the event, then the inclusion/exclusion criteria will be amended to exclude this ’at risk’ group.

The protocol procedures may be revised including but not limited to the duration of therapy. The study may also be stopped. See Table 2.

**Table 2:** Summary of adverse events

Adverse events	Non-device-related	Device-related	
Non-serious	Adverse event (AE)	Adverse device effect (ADE)	
Serious		Serious adverse device effect (SADE)	
		Anticipated	Unanticipated
		Anticipated serious adverse device effect (ASADE)	Unanticipated serious adverse effect (USADE)

### Causality and adverse device effects

The relationship of both AEs and SAEs to the study device will be determined as follows (Table 3).

**Table 3:** Causality of adverse events

Causality	Definition
Almost certainly	Starts within a time related to the operation of the device, and No obvious alternative medical explanation
Probably	Starts within a time related to the operation of the device, and Cannot be reasonably explained by known characteristics of the patient’s clinical state
Possibly	Starts within a time related to the operation of the device, and A causal relationship between the operation of the device and the adverse event is at least a reasonable possibility
Unrelated	The AE is definitely not associated with the study device

The Investigator must endeavour to obtain sufficient information to determine the causality of the AE and must provide his/her opinion of the causal relationship between each AE and the study device. This may require instituting supplementary investigations of significant AEs based on their clinical judgement of the likely causative factors and/or include seeking a further opinion from a specialist in the field of the AE.

### Adverse event and device deficiency reporting

All AEs during the study will be reported on the Adverse Event page of the Case Report Form (CRF). If events are deemed serious, a separate Serious Adverse Event form will also be completed.

Device deficiencies during the study will be recorded on a dedicated form. The Principal Investigator will record the action taken to alleviate the AE/SAE. AEs may be recorded as ongoing at the end of the study, but SAEs will be followed up until resolved. Trained medical personnel will be accessed to act with appropriate diagnostic and therapeutic measures until the subject has recovered. SAEs will be reported to the Principal Investigator within 24 hours.

### Withdrawals and dropouts

During the study, treatment may be discontinued for many reasons such as the occurrence of a disease, an AE that could interfere with the subject’s evaluation or simply upon the subject’s request to discontinue for any reason. Concurrent medical events that do not interfere with scheduled testing and that are judged by the Investigator to not have an effect on the outcome measures will not disqualify a subject from continuing in the study. If a subject is withdrawn from the study because of an AE, treatment discontinuation must be explained on the Adverse Event page of the CRF.

Patients will be advised that they are free to withdraw from the study at any time for any reason or, if necessary, the Investigator may withdraw a subject from the study to protect the subject’s health. The Investigator may withdraw a subject from the study if it is considered that the scientific, and therefore, ethical standards of the study are compromised. Patients may also be withdrawn for not complying with study procedures. The type and timing of the withdrawal for withdrawal will be fully recorded on the CRF. Should a patient choose to withdraw from treatment, they will be asked if they are still available for follow-up data collection.

### Trial exit

Participants will exit the trial completely if:

- They have completed their participation
- They request to/are unable to continue being followed up
- They experience an AE (if the AE is deemed due to trial involvement) such that they are unable to complete follow-up
- They die

### Overall timescale for the study

Recruitment began as soon as all necessary approvals (REC, Medicines and Healthcare products Regulatory Agency (MHRA) and Trust R&D) were obtained. The intention is that recruitment will run until a power calculation of participant number is reached. A power calculation will be performed from ongoing cohort study and existing evidence with a maximum of 100 participants in total. Patients are involved in the trial for 12 weeks only.

The outline of events is given in Table 4.

**Table 4:** Standard Protocol Items: Recommendations for Interventional Trials (SPIRIT) diagram of events timeline

	Study period						
	Enrolment and screening	Allocation	Post randomisation				Exit
Timepoint			Treatment period	4/52 F/U	8/52 F/U	12/52 F/U	
Enrolment:							
Eligibility screen	X						
Demographics	X						
Past medical history	X						
Provision of Patient Information Sheet	X						
Informed consent		X					
Randomisation/allocation		X					
Interventions:							
Active shockwave treatment (Group A)			9 sessions (3 × per week for 3 weeks)				
Sham treatment (Group B)			9 sessions (3 × per week for 3 weeks)				
Assessments:							
Ankle-brachial pressure index	X			X	X	X	
Claudication distance	X			X	X	X	
Maximum walking distance	X			X	X	X	
SF-36 questionnaire	X			X	X	X	
EQ-5D questionnaire	X			X	X	X	
Patient tolerability VAS			Recorded after each treatment				
Adverse events (bruising, bleeding, etc*.*)			Recorded after each treatment	X	X	X	

### Study completion

Completion refers to the date of final data collection from the last patient. Paper records from the trial will be stored for 5 years from trial end.

Data will be collected and collated using a specifically designed database which will be kept on hospital central servers on a limited access hard drive. Access will be via password-protected log-in on hospital servers only and will be limited to members of the Academic Vascular Surgery Unit. The file itself will have password-protected opening. Electronic records will be stored for 5 years from trial end.

### Data management

The use and control of all data will comply at all times with the requirements of the Data Protection Act 1998. The Principal Investigator of the study will register with the local Data Protection Officer. All data management procedures will be detailed and referred to as the Data Management Plan. All data will be entered and checked by a second person.

The database will be designed and built by the research team using Microsoft Excel. Appropriate CRFs will be prepared for the collection of the data requested by the protocol. All response variables will be entered into the database by the data management personnel.

### Statistical considerations

Statistical advice has been sought from the Statistical Consultancy Service (Hull York Medical School, University of York) in preparing this protocol. Further review will be sought when all results have been collected and the analysis performed.

Data will be analysed as intention to treat. The SPSS computer package will be used with a *p* value of <0.05 taken as the level of significance.

#### Sample size calculation

A power calculation will be performed from the ongoing cohort study and existing evidence with a maximum of 100 participants in total.

#### Analysis

Data will be analysed on an intention-to-treat basis. The main aim is to provide estimates of distribution and variability of outcomes with comparisons made between the shockwave treatment group and sham group. These will be expressed principally as means or medians and confidence intervals.

### Monitoring and quality assurance

The Principal Investigator is responsible for the day-to-day monitoring and management of the study. The Research Office, on behalf of the Sponsor, will monitor and conduct random audits on a selection of studies in its clinical research portfolio. Monitoring and auditing will be conducted in accordance with the Department of Health Research Governance Framework for Health and Social Care (April 2005), and in accordance with the Sponsor’s monitoring and audit policies and procedures.

The organisation, monitoring and quality assurance of this clinical study are the responsibility of the Sponsor and the Principal Investigator. To ensure the accuracy of data, direct access to source documents by the representatives of both the Sponsor and regulatory authorities is mandatory. Anonymity of the subjects will be maintained at all times.

The investigator(s)/institution(s) will permit study-related monitoring, audits, REC review and regulatory inspection(s), providing direct access to source data/documents. Patient consent to this is specifically sought in the Informed Consent Form.

### Study completion or discontinuation

Upon completion of the study, the following activities, when applicable, must be conducted by the Investigator and by the Sponsor’s Monitor, as appropriate:

- Data clarifications and/or resolutions
- Review of site study records for completeness

In addition, the Sponsor, the Funder and the Principal Investigator reserve the right to temporarily suspend or prematurely discontinue this study for any reason. If such action is taken, the Sponsor will discuss this with the Funder and the Investigator (including the reasons for taking such action) at that time.

If the study is terminated for safety reasons, the Sponsor will promptly inform the regulatory authorities of the suspension or termination of the study and the reason(s) for the action. The Investigator must inform the REC promptly and provide the reason for the suspension or termination. After such a decision, the Investigator must call in all participating patients within a reasonable time period. At this visit all medical files and CRFs must be completed as far as possible.

### Administrative procedures

#### Ethical considerations

This protocol has been prepared in accordance with the Standard Protocol Items: Recommendations for Interventional Trials (SPIRIT) checklist. See Additional file 1.

This study is conducted in accordance with the principles of the Declaration of Helsinki (Ethical Principles for Medical Research Involving Human Subjects).

#### Participant data protection

Participants will be informed that their data are held on file and that these data may be viewed by the Sponsor and by external auditors on behalf of either the Sponsor or regulatory agencies. Participants will similarly be informed that these data and a report of the study will be submitted to the Sponsor and may also be submitted to government agencies and perhaps for publication, but that they will only be identified in such reports by their study identification number, initials and perhaps their gender and age. The investigators undertake to hold all personal information in confidence and in compliance with the Data Protection Act 1998 and Caldicott Committee policies.

Data will be collected and collated using the database and data analysis software SPSS. This will be kept on hospital central servers on a limited access hard drive. Access will be via password-protected log-in on hospital servers only and will be limited to members of the Academic Vascular Surgery Unit. The file itself will have password-protected opening.

#### Data handling and record keeping

The Principal Investigator will be responsible for data collection, recording and quality. Electronic data will be stored on a National Health Service (NHS) Trust computer within the vascular laboratory. The IT services department has a backup procedure approved by auditors for disaster recovery. Servers are backed up to tape media each night; the tapes run on a 4-week cycle. Files stay on the server unless deleted by accident or deliberately. Anything deleted more than 4 weeks previously is therefore lost. Additional archive backups are taken for archived data, so data should not be lost from this type of system, e.g. with the use of FileVision, which stores medical records. Tapes are stored in a fireproof safe. Study documents (paper and electronic) will be retained in a secure location and kept locked when not in use during the trial and after it has finished. All essential documents including source documents will be retained for a minimum period of 5 years after study completion (last visit of the last patient). A label stating the date after which the documents can be destroyed will be placed inside the front cover of the case notes of trial participants. Data will be collected and retained in accordance with the Data Protection Act 1998.

#### Indemnity

This is an NHS-sponsored research study. If there is negligent harm during the clinical trial when the NHS body owes a duty of care to the person harmed, NHS indemnity covers NHS staff and medical academic staff with honorary contract only when the trial has been approved by the Hull and East Yorkshire R&D department. NHS indemnity does not offer no-fault compensation and is unable to agree in advance to pay compensation for non-negligent harm. The University of Hull has an insurance policy that includes cover for no-fault compensation in respect of accidental injury to a research subject.

## Discussion

There is significant research suggesting a neovascularisation/angiogenic response to ESWT when applied to soft tissue. However, only several small, low-level research trials have investigated the use of ESWT in patients with peripheral arterial disease and, more specifically, intermittent claudication. Non-invasive management of patients suffering from intermittent claudication consists of best medical treatment plus supervised exercise programmes (SEPs). Unfortunately, uptake and adherence to SEPs is extremely poor in most centres; therefore, there is potential for an alternative non-invasive treatment option. The purpose of this study is to firstly investigate whether this treatment is safe and well tolerated by patients when applied to the calf muscle bulk, and also to study whether the angiogenic response seen in soft tissue research relating to ESWT has a positive effect on this cohort of patients and whether it is significant enough to improve clinical outcomes such as maximum walking distance, pain-free walking distance and ankle-brachial pressure index.

### Trial status

This trial remains in the recruitment phase.

## Additional file

Additional file 1: SPIRIT checklist. (DOC 121 kb)

## Abbreviations

ABPI: Ankle-brachial pressure index
BMI: Body mass index
BMT: Best medical treatment
CI: Confidence interval
CILLI: Clinical indicator of lower limb ischaemia
CLI: Critical limb ischaemia
CRP: C-reactive protein
ESWT: Extracorporeal shockwave therapy
GP: General practitioner
HDL: High-density lipoprotein
IC: Intermittent claudication
ICD: Intermittent claudication distance
NICE: National Institute of Health and Care Excellence
nM: Nanomole
P: Probability
PABPI: Post exercise ankle-brachial pressure index
PAD: Peripheral arterial disease
PF: Physical function
PRD: Patient reported walking distance
PTA: Percutaneous transluminal angioplasty
QoL: Quality of life
SEP: Supervised exercise programme
SF: Social function
SF-36: Short Form 36
TASC: Trans-Atlantic Inter-Society Consensus
vLDL: Very low-density lipoprotein
vWF: Von Willebrand factor
